# A Novel Dibenzoxazepine Attenuates Intracellular Salmonella Typhimurium Oxidative Stress Resistance

**DOI:** 10.1128/Spectrum.01519-21

**Published:** 2021-12-01

**Authors:** Cheng-Yun Hsu, Yi-Lun Wu, Hsueh-Chun Lin, Man-Yi Lin, Shih-Hsiu Chou, Chung-Wai Shiau, Hao-Chieh Chiu

**Affiliations:** a Department of Clinical Laboratory Sciences and Medical Biotechnology, College of Medicine, National Taiwan University, Taipei, Taiwan; b Institute of Biopharmaceutical Sciences, National Yang Ming Chiao Tung University, Taipei, Taiwan; c Department of Laboratory Medicine, National Taiwan University Hospital, College of Medicine, National Taiwan University, Taipei, Taiwan; University of Florida

**Keywords:** antipsychotics, high-content assay, loxapine, ROS

## Abstract

Salmonella enterica serovar Typhimurium is the leading cause of invasive nontyphoidal salmonellosis. Additionally, the emergence of multidrug-resistant S. Typhimurium has further increased the difficulty of controlling its infection. Previously, we showed that an antipsychotic drug, loxapine, suppressed intracellular Salmonella in macrophages. To exploit loxapine’s antibacterial activity, we simultaneously evaluated the anti-intracellular Salmonella activity and cytotoxicity of newly synthesized loxapine derivatives using an image-based high-content assay. We identified that SW14 exhibits potent suppressive effects on intramacrophagic S. Typhimurium with an 50% effective concentration (EC_50_) of 0.5 μM. SW14 also sensitized intracellular Salmonella to ciprofloxacin and cefixime and effectively controlled intracellular multidrug- and fluoroquinolone-resistant S. Typhimurium strains. However, SW14 did not affect bacterial growth in standard microbiological broth or minimal medium that mimics the phagosomal environment. Cellular autophagy blockade by 3-methyladenine (3-MA) or shATG7 elevated the susceptibility of intracellular Salmonella to SW14. Finally, reactive oxygen species (ROS) scavengers reduced the antibacterial efficacy of SW14, but the ROS levels in SW14-treated macrophages were not elevated. SW14 decreased the resistance of outer membrane-compromised S. Typhimurium to H_2_O_2_. Collectively, our data indicated that the structure of loxapine can be further optimized to develop new antibacterial agents by targeting bacterial resistance to host oxidative-stress defense.

**IMPORTANCE** The incidence of diseases caused by pathogenic bacteria with resistance to common antibiotics is consistently increasing. In addition, Gram-negative bacteria are particularly difficult to treat with antibiotics, especially those that can invade and proliferate intracellularly. In order to find a new antibacterial compound against intracellular Salmonella, we established a cell-based high-content assay and identified SW14 from the derivatives of the antipsychotic drug loxapine. Our data indicate that SW14 has no effect on free bacteria in the medium but can suppress the intracellular proliferation of multidrug-resistant (MDR) S. Typhimurium in macrophages. We also found that SW14 can suppress the resistance of outer membrane compromised Salmonella to H_2_O_2_, and its anti-intracellular Salmonella activity can be reversed by reactive oxygen species (ROS) scavengers. Together, the findings suggest that SW14 might act via a virulence-targeted mechanism and that its structure has the potential to be further developed as a new therapeutic against MDR Salmonella.

## INTRODUCTION

Salmonella enterica, a Gram-negative facultative intracellular bacterium, is the most common cause of gastrointestinal infections (1). Among the 2,600 serotypes identified, S. enterica serovar Typhimurium (here S. Typhimurium) often causes localized gastroenteritis in humans and severe typhoid-like disease in mice (2). However, in immunocompromised patients, S. Typhimurium infection can lead to the fatal disease invasive nontyphoidal salmonellosis (iNTS), which caused approximately 77,500 deaths in 2017 (3). In addition, the emergence of multidrug-resistant (MDR) and fluoroquinolone-resistant strains of S. enterica has further dampened its treatment, leading to higher rates of morbidity and mortality (4). Thus, the World Health Organization (WHO) has listed antibiotic-resistant S. enterica as one of the pathogens for which there is a great need for new antibiotic treatment (5).

During infection, S. Typhimurium can express various virulence factors to invade host cells and proliferate in a phagosome-like vacuole called the Salmonella-containing vacuole (SCV) (6). In contrast, host cells also possess diverse innate defenses to control intracellular Salmonella infection (7–9). For example, macrophages can exploit multisubunit NADPH-dependent phagocytic oxidase (Phox or NOX2) to generate reactive oxygen species (ROS) as a key defense mechanism against invading bacteria (10). NOX2 assembles on the phagolysosomal membrane, reducing oxygen to produce superoxide anions, which are then enzymatically or spontaneously reduced to different kinds of ROS, including hydrogen peroxide and hydroxyl radicals. All of these ROS can control invasive microorganisms, but the detailed mechanisms have not been completely clarified (11). In addition, intracellular Salmonella has been shown to be controlled by autophagy, an intracellular degradation system (12). The induction of autophagy can reduce the intracellular bacterial load in macrophages and prolong the survival of Salmonella-infected mice (13, 14).

Dibenzoxazepines are a class of antipsychotic drugs with a mechanism of action that is similar to that of phenothiazines. Previously, we found that the dibenzoxazepine antipsychotic drug loxapine exhibits suppressive activity against S. Typhimurium and other pathogenic bacteria in macrophages (15). To exploit the antibacterial activity of loxapine, we synthesized a series of loxapine derivatives and screened their anti-intracellular Salmonella activity and cytotoxicity. Our efforts led to the identification of a novel small-molecule antibacterial agent, SW14, that can effectively restrict the intracellular proliferation of S. Typhimurium at submicromolar concentrations but did not inhibit bacterial growth in broth at concentrations up to 64 μM, suggesting that it acts via a virulence-targeted or host-targeted mechanism. Accordingly, the mode of action of SW14’s antibacterial activity against intracellular S. Typhimurium, including MDR strains, is the focus of this investigation.

## RESULTS

### Identification of a potent loxapine derivative.

**FIG 1:**
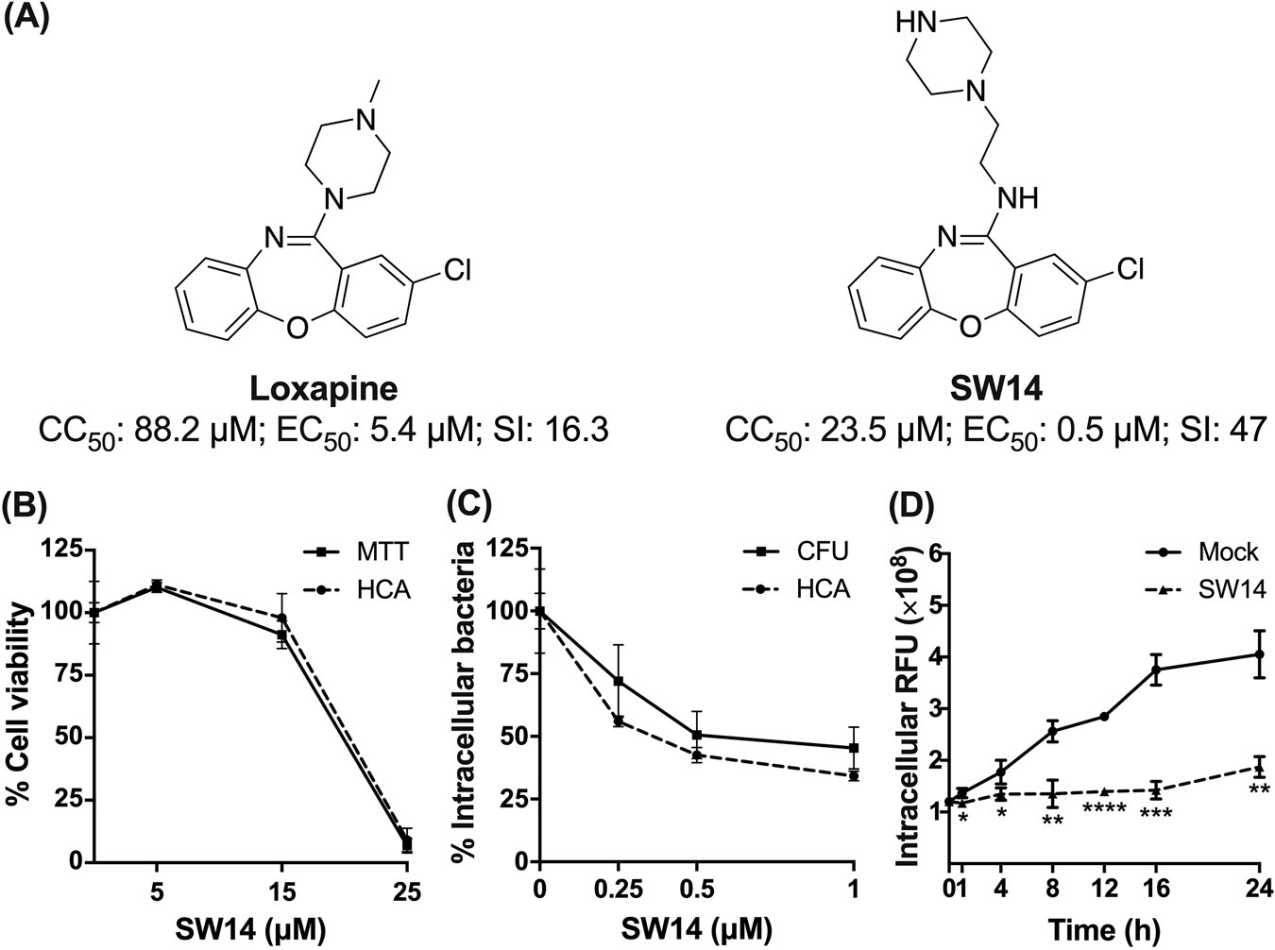
SW14 suppresses the intracellular replication of S. Typhimurium in macrophages. (A) Structures, 50% cytotoxic concentration (CC_50_) toward macrophages, 50% effective concentration (EC_50_) against intracellular Salmonella, and selectivity index (SI; CC_50_/EC_50_) of loxapine and SW14. The data are representative of two independent experiments. (B) RAW264.7 cells were treated with various doses of SW14, and cell viability was evaluated using HCA and an 3-(4,5-dimethylthiazol-2-yl)-2,5-diphenyl-2H-tetrazolium bromide (MTT) cell viability assay. The data are expressed as percentages relative to the untreated control and are presented as the means ± SD (*n* = 3/group). (C) S. Typhimurium-infected RAW264.7 cells were treated with a range of SW14 doses in the presence of gentamicin (20 mg/L) for 24 h. Intracellular bacteria survival was assessed using HCA and a CFU assay. The data are expressed as percentages relative to the untreated control and are presented as the means ± SD (*n* = 3/group). (D) S. Typhimurium-infected RAW264.7 cells were treated with 0.5 μM SW14 in combination with gentamicin (20 mg/L). The relative number of intracellular bacteria was determined at designed times using HCA for a total of 24 h, and the results are expressed as relative fluorescence units (RFU). The data are presented as the means ± SD (*n* = 3/group). *, *P < *0.05; **, *P < *0.01; ***, *P < *0.001; ****, *P < *0.0001.

Our previous efforts to discover nonantibiotic drugs with antibacterial properties led to the determination that loxapine, an antipsychotic drug, possesses antibacterial activity against intracellular S. Typhimurium in macrophages (15). To further exploit the antibacterial activity of loxapine, we synthesized a series of loxapine derivatives and utilized image-based high-content analysis (HCA) to simultaneously evaluate their antibacterial activity by assessing the number of intracellular S. Typhimurium on the basis of red fluorescent protein (RFP) signals and cytotoxicity toward infected murine macrophage RAW264.7 cells via nuclear staining (data not shown). Among the newly synthesized derivatives, SW14 exhibited the most potent antibacterial activity against intracellular S. Typhimurium (50% effective concentration [EC_50_] = 0.5 μM) with no obvious toxicity toward the host cells (50% cytotoxic concentration toward cells [CC_50_] = 23.5 μM), resulting in a selectivity index (CC_50_/EC_50_) nearly three times higher than that of loxapine (Fig. 1A and Fig. S1). The anti-intracellular Salmonella activity and cytotoxicity of SW14 were further confirmed by a CFU assay and 3-(4,5-dimethylthiazol-2-yl)-2,5-diphenyl-2H-tetrazolium bromide (MTT) cell viability assay, respectively. Consistent with the findings of HCA, SW14 exhibited a potent suppressive effect on the viability of intracellular Salmonella at submicromolar concentrations, indicating that the anti-intracellular Salmonella activity of SW14 observed from the HCA was not due to interference with the RFP expression (Fig. 1B and C). Moreover, closer time-dependent examination of the effects of SW14 on intracellular Salmonella showed that there was a significant difference in the relative number of bacteria in the infected cells after treatment with SW14 for 1 h, and the number of intracellular bacteria did not increase significantly in the subsequent time period (Fig. 1D), suggesting that SW14 acts by limiting the proliferation of intracellular Salmonella.

### SW14-sensitized intracellular Salmonella to ciprofloxacin and cefixime.

**FIG 2:**
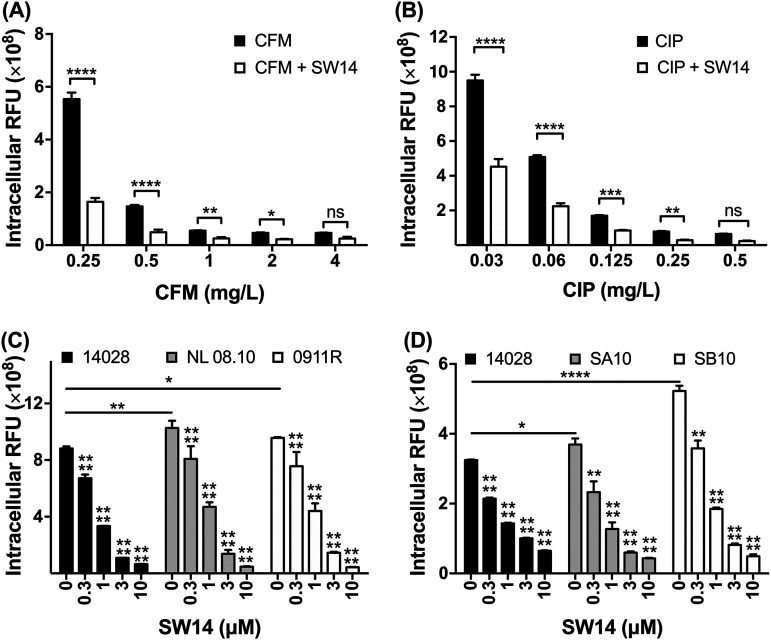
SW14 is effective against multiple antibiotic-resistant S. Typhimurium strains in macrophages. (A, B) Salmonella-infected RAW264.7 cells were treated with various concentrations of cefixime (CFM; A) and ciprofloxacin (CIP; B) alone or in combination with 0.5 μM SW14. After 24 h, the relative numbers of intracellular bacteria were determined using HCA, and the results are expressed as RFUs. The data are presented as the means ± SD (*n* = 3/group). *, *P < *0.05; **, *P < *0.01; ***, *P < *0.001; ****, *P < *0.0001. (C, D) RAW264.7 cells were infected with multidrug-resistant isolates 0911R and NL08.10 (C) or ciprofloxacin-resistant strains SA10 and SB10 (D) of S. Typhimurium followed by treatment with various doses of SW14 combined with 20 mg/mL gentamicin for 24 h. The relative numbers of intracellular bacteria were determined using HCA, and the results are expressed as relative fluorescence units (RFU). The data are presented as the means ± SD (*n* = 3/group). *, *P < *0.05; **, *P < *0.01; ****, *P < *0.0001.

Currently, the first-line antibiotics for the treatment of Salmonella infections are fluoroquinolones and cephalosporins (16). To observe whether SW14 can increase the sensitivity of intracellular Salmonella to these two classes of antibiotics, we infected RAW264.7 cells with RFP-expressing Salmonella and treated the infected cells with ciprofloxacin, cefixime, or combinations of each antibiotic and SW14 for 24 h. Our results indicated that ciprofloxacin and cefixime inhibited the survival of intracellular Salmonella in macrophages in a dose-dependent manner (Fig. 2A and B). Moreover, the inhibitory activity of these two antibiotics was enhanced when they were used in combination with 0.5 μM SW14, as demonstrated by the fact that the relative number of intracellular bacteria in cells receiving combination treatment was significantly reduced to the same level as that of cells treated with the corresponding antibiotic at a 2-fold concentration (Fig. 2A and B). Therefore, our results indicate that SW14 can enhance the suppression caused by these first-line antibiotics against intracellular Salmonella.

### SW14 is active against multidrug- and fluoroquinolone-resistant S. Typhimurium in macrophages.

The emergence and spread of S. Typhimurium that are resistant to ampicillin, chloramphenicol, streptomycin, sulfamethoxazole, and tetracycline (ACSSuT type) are a serious threat to public health (17, 18). To investigate whether SW14 can inhibit drug-resistant Salmonella infection in cells, we transformed the RFP-expressing plasmids into two MDR clinical isolates of S. Typhimurium and evaluated the effects of SW14 on infection from these two MDR isolates in macrophages. Compared to the reference S. Typhimurium strain, the two MDR S. Typhimurium isolates were highly resistant to most of the antibiotics tested (Table S1). Additionally, the relative numbers of bacteria in cells infected with the MDR strains were higher than that of cells infected with the reference strain. Despite differences in antibiotic resistance and infectivity, the intracellular replication of the two MDR S. Typhimurium isolates remained susceptible to the suppressive activity of SW14 (Fig. 2C). Next, we extended the investigation to S. Typhimurium isolates with resistance to ciprofloxacin, the first-line antibiotic for salmonellosis. Similar to the MDR clinical isolates, the ciprofloxacin-resistant strain showed higher infectivity than the reference strain. However, the intracellular proliferation of these ciprofloxacin-resistant isolates was still highly sensitive to SW14 (Fig. 2D). These results indicate that the antibacterial activity of SW14 is not affected by bacterial infectivity or resistance mechanisms to common antibiotics.

### Autophagy is not involved in the anti-intracellular Salmonella activity of SW14.

**FIG 3:**
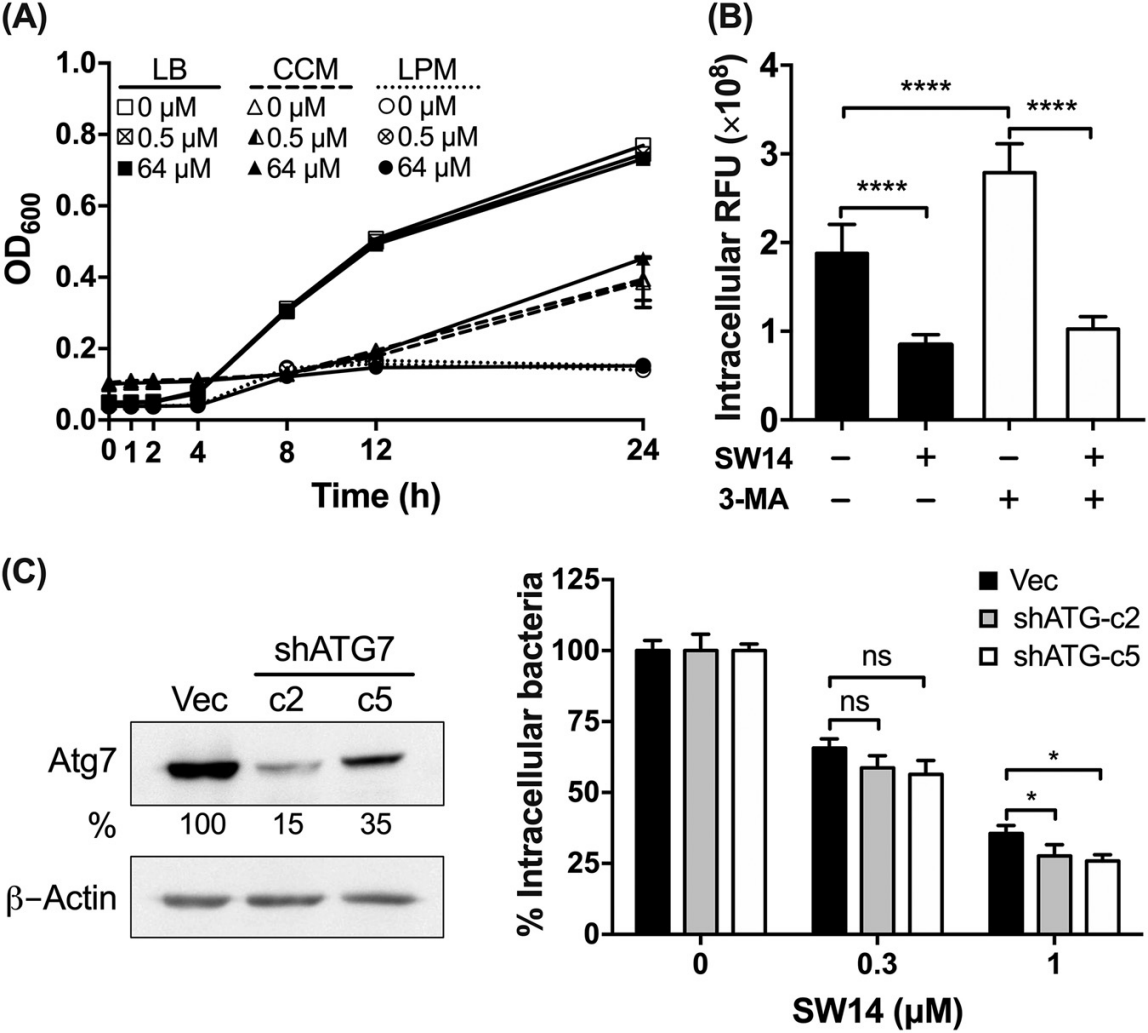
The antibacterial activity of SW14 is not mediated by suppressing bacterial growth or cellular autophagic defense. (A) The growth of S. Typhimurium in LB medium, cell culture medium (CCM), or LPM medium containing various concentrations of SW14 was monitored by measuring the OD_600_ of each bacterial culture at designated times for a total of 24 h. The data are presented as the means ± SD (*n* = 3/group). (B) Infected RAW264.7 cells were treated with 3-MA (10 mM) for 1 h followed by SW14 (0.5 μM) or mock (DMSO) treatment for 24 h. The viability of the intracellular bacteria was assessed using HCA, and the results are expressed as percentages relative to the mock-treated cells. The data are presented as the means ± SD (*n* = 3/group). ****, *P < *0.0001. (C) RAW264.7 cells were stably transfected with a plasmid to express shRNA targeting Atg7 (shATG7) or with the empty vector (Vec) and then infected with S. Typhimurium followed by treatment with various concentrations of SW14 and 20 mg/mL gentamicin for 24 h. The numbers of surviving intracellular bacteria were determined by HCA, and the results are expressed as a percentage of the control group. The left panel shows the levels of the Atg7 protein in the stable transfectants as determined by immunoblotting. Bands for Atg7 were quantified by densitometry and normalized to those of β-actin. Percentages represent the relative expression levels of Atg7 in the shATG7 transfectants compared to the vector transfectants. The data are representative of two independent experiments. The right panel shows intracellular bacterial survival in SW14-treated RAW264.7 cells with shRNA-mediated knockdown of Atg7 expression. The data shown are the means; error bars represent the SD (n = 3). *, *P < *0.05 for the differences between the drug-treated groups and their respective control groups. LPM, low-phosphate, low-magnesium medium; 3-MA, 3-methyladenine; ns, nonsignificant. OD_600_, optical density at 600 nm.

To elucidate the mechanism of action of SW14, we first examined whether SW14 can directly inhibit bacterial growth in medium by exposing free bacteria to escalating concentrations of SW14 in Luria-Bertani (LB) medium and cell culture medium (CCM; Dulbecco’s modified Eagle’s medium [DMEM] supplemented with 10% fetal bovine serum [FBS]) followed by monitoring the optical density at 600 nm (OD_600_) of the bacterial cultures at different time points for 24 h. As shown in Fig. 3A, SW14 did not show significant inhibitory effects on the growth of bacteria in either medium at concentrations up to 64 μM. Next, we further tested the effects of SW14 on the growth of Salmonella in acidic low-phosphate, low-magnesium (LPM) medium, which mimics the environment of macrophage phagosomes (19). Consistent with previous reports that showed that the outer membrane of Salmonella is compromised in the phagosome microenvironment, the susceptibility of Salmonella to several antibiotics was elevated in the LPM medium (Table S2). However, we were still unable to detect any significant change in the growth of Salmonella in SW14-containing LPM medium. These findings suggest that SW14-induced suppression of intracellular bacteria could be mediated by a host- or virulence-directed mechanism.

Autophagy was originally characterized as a cellular response to stress, but now it has also been considered to be a defense mechanism to combat host cell infection by intracellular Salmonella (12). Previously, Conway et al. showed that trifluoperazine, an antipsychotic drug with a structure similar to that of loxapine, can suppress intracellular Salmonella via the induction of autophagy (20). Accordingly, we first evaluated the role of autophagy in SW14’s anti-intracellular Salmonella activity by treating infected cells with 3-methyladenine (3-MA), an autophagy inhibitor, to block cellular autophagy followed by treatment with SW14 for 24 h. As shown in Fig. 3B, the relative number of intracellular bacteria increased significantly in 3-MA-treated cells, suggesting that autophagic defense was inhibited. Compared to mock-treated cells, the viabilities of intracellular Salmonella in cells treated with SW14 or the combination of 3-MA and SW14 were 45 and 36.8%, respectively. Next, we further employed shRNA to knockdown the expression of Atg7, a key protein involved in autophagosome formation, and obtained two clones of RAW264.7 cells with Atg7 levels of 15 and 35% that of the cells transfected with empty vector (Fig. 3C, *left*). Similar to the findings of 3-MA-mediated blockade of the autophagy pathway, knockdown of Atg7 in RAW264.7 cells also rendered intracellular Salmonella more susceptible to the suppressive effects of SW14 (Fig. 3C, *right*). Our results indicated that autophagy did not contribute to the SW14-mediated suppression of intracellular Salmonella.

### SW14 attenuates bacterial resistance to oxidative stress.

**FIG 4:**
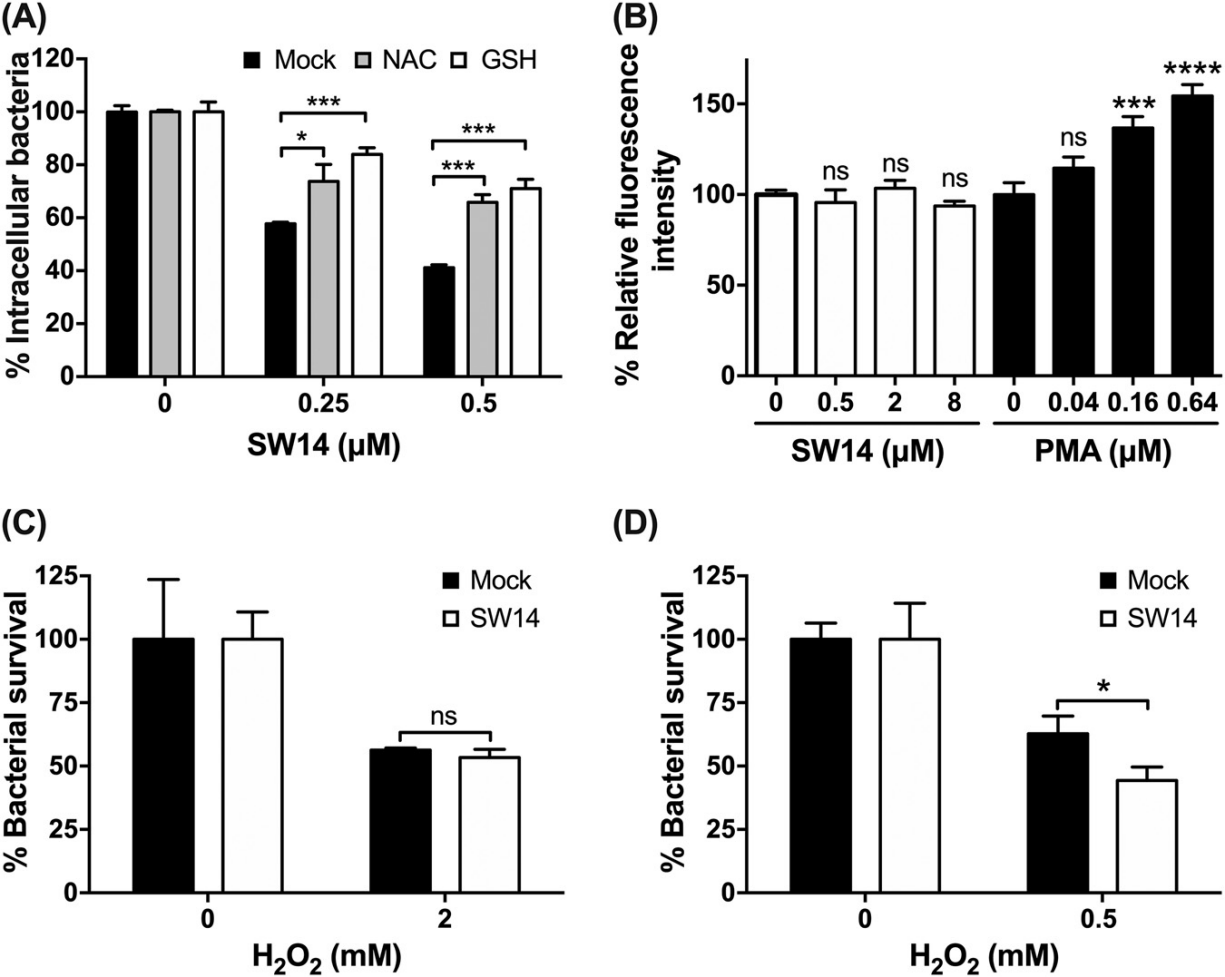
SW14 sensitizes outer membrane-compromised S. Typhimurium to oxidative stress. (A) ROS scavengers reversed the suppressive effects of SW14 on intracellular S. Typhimurium. The viabilities of intracellular Salmonella in RAW264.7 cells treated with SW14 with/without the ROS scavengers *N*-acetylcysteine (NAC; 10 mM) and glutathione (GSH; 10 mM) were assessed using HCA. The data are presented as the means ± SD (*n* = 3/group). *, *P < *0.05; ***, *P < *0.001. (B) SW14 did not induce ROS production in macrophages. Salmonella-infected RAW264.7 cells were loaded with 25 μM H_2_DCFDA for 45 min followed by treatment with SW14 or PMA at various concentrations. The data are expressed as percentages relative to the untreated control and are presented as the means ± SD (*n* = 3/group). ns, nonsignificant; ***, *P < *0.001; ****, *P < *0.0001. (C, D) S. Typhimurium ATCC 14028 was treated with H_2_O_2_ alone or in combination with SW14 (0.5 μM) in LB (C) or LPM (D) for 1 h followed by evaluation by the CFU assay to determine bacterial viability. The data are expressed as percentages relative to the number of bacteria in H_2_O_2_-free medium and are presented as the means ± SD (*n* = 3/group). ns, nonsignificant; *, *P < *0.05.

Oxidative stress is another innate defense system in host cells that controls intracellular Salmonella infection (21). We found that the suppressive effects of SW14 against S. Typhimurium within macrophages were reversed in the presence of two ROS scavengers: *N*-acetylcysteine (NAC) and glutathione (GSH) (Fig. 4A). To establish whether SW14’s anti-intracellular Salmonella activity was mediated by ROS induction, we loaded Salmonella-infected RAW264.7 cells with the ROS indicator 2',7'-dichlorodihydrofluorescein diacetate (H_2_DCFDA) and treated cells with SW14 or phorbol 12-myristate 13-acetate (PMA), a known ROS inducer (22), for 1 h. As shown in Fig. 4B, PMA significantly elevated ROS levels in infected macrophages, as demonstrated by the increase in the fluorescence signal of H_2_DCFDA. In contrast, we did not observe any significant change in the H_2_DCFDA signal in SW14-treated cells at concentrations up to 8 μM, which is 16 times greater than its EC_50_ against intracellular Salmonella (Fig. 4B). Next, we investigated the effects of SW14 on bacterial resistance to ROS and found that the susceptibility of S. Typhimurium to H_2_O_2_ was significantly increased after SW14 treatment at its EC_50_ concentration (0.5 μM) in minimal LPM medium but not in nutrient-rich LB broth (Fig. 4C and D). Thus, these findings implicated that SW14 might suppress the resistance of Salmonella to oxidative stress under conditions that compromise the outer membrane.

## DISCUSSION

The rapid emergence of antibiotic-resistant pathogenic bacteria highlights the urgent need for an innovative strategy to combat bacterial infections. Among the newly proposed strategies, the virulence-targeted method is particularly attractive. Unlike common antibiotics that kill or inhibit bacterial growth, virulence-targeted interventions control bacterial infection by suppressing the bacterial pathogenic factors important for infection. Due to their unique mechanism of action, virulence-targeted agents exert lower selective pressure and are less likely to induce resistance. In this study, we discovered a novel loxapine derivative, SW14, that displays great potency in controlling intracellular Salmonella proliferation but has no suppressive effect on the growth of free bacteria in the extracellular environment. Moreover, SW14 is active against both ACSSuT-type MDR isolates and ciprofloxacin-resistant strains of S. Typhimurium. Subsequent mechanistic studies indicated that host ROS play an important role in SW14’s antibacterial activity and that SW14 can attenuate bacterial resistance to oxidative stress, suggesting that it acts via a virulence-targeted mechanism.

In response to bacterial invasion, mammalian cells have evolved a variety of defense mechanisms, including autophagy and ROS, to control infection. These innate immune responses can interact with each other; for example, inhibition of autophagy by 3-MA can increase intracellular ROS levels (23). Given that SW14 can reduce bacterial resistance to oxidative stress, 3-MA-induced ROS generation further enhanced the inhibitory effect of SW14 on intracellular Salmonella (Fig. 4B). In contrast, intracellular bacteria also employ numerous mechanisms to evade the damaging effects of host ROS. These mechanisms include the direct detoxification of ROS by enzymes, including superoxide dismutases (SODs), catalases, and peroxidases. In addition, bacterial efflux pumps contribute to bacterial resistance to ROS (21). For instance, the natural substrate of MacAB, an ABC-type multidrug efflux system in Salmonella, can offer bacterial resistance to oxidative stress (24). Impairment of MacAB reduced bacterial virulence in a BALB/c mouse salmonellosis model (25). Another efflux pump, the RND-type AcrAB efflux system, is also involved in the intracellular survival of S. Typhimurium, and suppression of its activity can reduce the intracellular bacterial load in macrophages (26). Previously, we observed that loxapine can suppress efflux pump activity in S. Typhimurium. A similar efflux pump suppressive effect on S. Typhimurium was also observed after SW14 treatment (data not shown). Currently, investigation of the interplay between SW14 and bacterial efflux systems is underway, which might provide information to guide the design of more potent virulence-targeted antibiotics against intracellular bacteria.

## MATERIALS AND METHODS

### Cells.

The RAW264.7 murine macrophage cell line was purchased from the Bioresource Collection and Research Center (BCRC) and maintained in DMEM (Gibco-BRL) supplemented with 10% heat-inactivated FBS (Gibco-BRL) at 37°C in a 5% CO_2_ atmosphere.

### Bacterial strains.

S. Typhimurium ATCC 14028 was obtained from the American Type Culture Collection (ATCC). Antibiotic-resistant isolates of S. Typhimurium (0911R, NL08.10, SA10, and SB10) were obtained from the Taiwan Centers for Disease Control. RFP-expressing S. Typhimurium was established by transforming pBR-RFP.1 (ampicillin-resistant) (12), pBR-RFP.1K (kanamycin-resistant), or pBR-RFP.1C (chloramphenicol-resistant) into bacteria. Bacteria were cultured in LB medium (Athena Enzyme Systems) at 37°C and stored in LB medium supplemented with 15% glycerol at −80°C.

### Reagents.

The starting materials for the synthesis of the loxapine-focused compound library were purchased from Sigma-Aldrich, Acros Organics, or Tokyo Chemical Industry (TCI). The synthetic details of the loxapine derivatives will be reported elsewhere. The purity of all tested compounds was determined to be >95% by proton nuclear magnetic resonance (^1^H NMR) spectra with Bruker DPX400 (400 MHz) instruments. Loxapine (Sigma-Aldrich), PMA (LC laboratories), H_2_DCFDA (Cayman Chemical), rifampicin (Bio Basic), SW14, and other loxapine derivatives were dissolved in dimethyl sulfoxide (DMSO; Sigma-Aldrich). Hoechst 33342 (Thermo Fisher Scientific), gentamicin (Bio Basic), ampicillin (United States Biochemical [USB]), ciprofloxacin (Sigma-Aldrich), streptomycin (Bio Basic), ofloxacin (Bio Basic), and vancomycin (Goldbio) were dissolved in deionized water and filtered through a 0.45-μm filter. Tetracycline (Bio Basic) and chloramphenicol (Amresco) were dissolved in 95% ethanol as stock solutions.

### Image-based high-content analysis of bacterial replication in macrophages.

Overnight cultures of RFP-expressing S. Typhimurium were prepared for infection by 1:100 dilution in fresh LB broth and incubation for 2 h at 37°C. The bacteria were then collected by centrifugation at 6,000 × *g* for 3 min and suspended in phosphate-buffered saline (PBS; pH 7.2) to an optical density of 0.6 at 600 nm, which was equivalent to 5 × 10^8^ CFU/mL. RAW264.7 macrophages were seeded in 96-well black clear-bottom plates (Greiner) and incubated for 20 h followed by infection with RFP-expressing S. Typhimurium at a multiplicity of infection (MOI) of 50 for 1 h. Infected macrophages were washed, exposed to 100 mg/L gentamicin for 1 h to eliminate extracellular bacteria, and treated with test compounds combined with gentamicin (20 mg/L), cefixime (0.25 to 4 mg/L), or ciprofloxacin (0.03 to 0.5 mg/L) for 24 h. Afterward, the cells were washed and stained with CellTracker Green (Thermo Fisher Scientific) for 30 min in serum-free DMEM followed by fixation in 3.7% formaldehyde (Sigma-Aldrich) for 20 min and stained with 0.2 mg/L DAPI (4′,6-diamidino-2-phenylindole; AAT Bioquest) for 30 min. Images of individual wells were captured and analyzed using a high-content imaging system (ImageXpress Micro 4; Molecular Devices). The CC_50_ and 50% effective concentration (EC_50_) values against intracellular bacteria of each compound were determined using CalcuSyn software (Biosoft).

### Analysis of bacterial growth in macrophages.

Overnight cultures of S. Typhimurium were diluted 1:100 in fresh LB broth and incubated for 2 h at 37°C. The bacteria were then collected and suspended in PBS to an OD_600_ of 0.6. RAW264.7 cells seeded in a 6-well plate were infected with S. Typhimurium at an MOI of 25 for 1 h. After infection, the cells were washed, exposed to 100 mg/L gentamicin for 1 h, and treated with SW14 (0.25 to 1 μM) in combination with 20 mg/L gentamicin for 24 h. Then, the infected cells were washed and lysed with 0.1% Triton X-100 in PBS for 10 min at 37°C to release the intracellular bacteria. The cell lysates were serially diluted in PBS, spread on LB agar plates, and incubated at 37°C for 18 h. The bacterial colonies on the plates were enumerated and expressed as CFU.

### Cell viability analysis.

The cells were seeded in 96-well plates (Greiner) and incubated for 20 h followed by treatment with test compounds for another 24 h. Then, the cells were treated with 0.5 g/L MTT and incubated at 37°C with 5% CO_2_ for 1 h. The culture media were removed, and the reduced MTT products were dissolved in DMSO. The absorbance at 570 nm was measured using a VersaMax Microplate reader (Molecular Devices). The CC_50_ of each compound was determined using CalcuSyn software.

### Bacterial growth assay.

Overnight-grown S. Typhimurium cultured in LB medium was inoculated into fresh cation-adjusted Mueller-Hinton broth (CAMHB; Becton, Dickinson and Company), DMEM supplemented with 10% FBS, or LPM medium (5 mM KCl, 7.5 mM (NH_4_)_2_SO_4_, 0.5 mM K_2_SO_4_, 10 mM glucose, 49 μM MgCl_2_, 337 μM PO_4_^3−^, 0.05% casamino acids, 80 mM MES, pH 5.8) (19) to a final concentration of 5 × 10^5^ CFU/mL, followed by exposure to different concentrations of SW14 in a flat-bottom 96-well plate. The plate was incubated at 37°C, and bacterial growth was monitored by measuring the absorbance at 600 nm with a VersaMax Microplate reader (Molecular Devices) at designated times for 24 h.

### MIC assay.

The minimal inhibitory concentration (MIC) of each test compound against the individual bacterial strains was determined by the broth microdilution method following the guidelines recommended by the Clinical and Laboratory Standards Institute (CLSI) (27). Briefly, overnight bacterial cultures in LB medium were inoculated into fresh CAMHB or LB medium to a final concentration of 5 × 10^5^ CFU/mL. Bacteria were then exposed to the test drugs at escalating concentrations, ranging from 0.125 to 64 mg/L, in triplicate in 96-well plates at 37°C for 24 h. The MIC of each individual compound was defined as the lowest concentration without visible bacterial growth.

### Knockdown of Atg7 expression in macrophages.

Knockdown of Atg7 expression was achieved by transfection of the macrophages with plasmids expressing short hairpin RNA (shRNA) against Atg7 (28). Three plasmids coding for different shRNA sequences were obtained from The RNAi Consortium (TRC) (29), National RNAi Core Facility, Academia Sinica, Taiwan, and prepared using an EndoFree plasmid purification kit (Qiagen). The cells were transfected with Lipofectamine 3000 (Thermo Fisher Scientific) according to the manufacturer’s instructions. Briefly, RAW264.7 cells were seeded in 6-well plates (2.5 × 10^5^ cells/well) for 24 h, and then the culture medium was replenished with fresh medium. The shRNA-expressing plasmids and Lipofectamine 3000 were complexed in Opti-MEM medium (Thermo Fisher Scientific) and then gently added to each well. Macrophages transfected with empty vector (pLKO_TRC005) served as controls. The cells were harvested at 48, 72, and 96 h after transfection, and the knockdown efficiency of each plasmid was assessed by immunoblotting with antibodies against Atg7 (GeneTex) and β-actin (GeneTex). The plasmid that caused the maximal repression of Atg7 was selected for subsequent experimentation. Stably transfected clones were selected using medium supplemented with puromycin (2.5 mg/L; Thermo Fisher Scientific), and immunoblotting was used to screen for knockdown of target gene expression. The selected clones were maintained in the presence of 1 mg/L puromycin to retain clonal homogeneity.

### Analysis of ROS in macrophages.

RAW264.7 macrophages were seeded in 96-well black plates and infected by S. Typhimurium ATCC 14028 at an MOI of 25 for 1 h. After infection, the cells were washed, exposed to 100 mg/L gentamicin for 1 h, washed with warm Krebs-Ringer phosphate buffer (KRP buffer; 120 mM NaCl, 5 mM KCl, 1.7 mM KH_2_PO_4_, 8.3 mM Na_2_HPO_4_, 10 mM glucose, 1 mM CaCl_2_, 1.5 mM MgCl_2_, pH 7.3) and stained with H_2_DCFDA at 37°C with 5% CO_2_ for 45 min. The cells were then washed with KRP buffer and treated with SW14 or PMA at the desired concentrations in KRP buffer with 10% FBS. Fluorescence signals at 488/525 nm (excitation/emission) were measured using a SpectraMax M5 Microplate Reader (Molecular Devices).

### Analysis of bacterial susceptibility to H_2_O_2_.

Overnight-grown S. Typhimurium cultured in LB medium were diluted 1:50 in LPM medium or LB medium followed by incubation at 37°C for 3 h. The bacterial suspension was adjusted to an OD_600_ of 0.2 in fresh LPM medium or LB medium, exposed to SW14 and H_2_O_2_ for 1 h at 37°C with agitation, then serially diluted 10-fold in PBS, and spread on LB agar plates. After incubation at 37°C for 18 h, the number of colonies was counted, and the results are expressed as CFU/mL.

### Statistical analysis.

The data are expressed as the means ± SD. Differences between group means were analyzed using multiple *t* tests or one-way analysis of variance (ANOVA) with Dunnett’s multiple-comparison test for independent samples. Differences were considered significant at a *P* value of <0.05. Statistical analyses were performed using GraphPad Prism (version 6.0; GraphPad Software).
