# Supplementary material for: A Novel Dibenzoxazepine Attenuates Intracellular Salmonella Typhimurium Oxidative Stress Resistance
Source: Microbiol Spectr. 2021 Dec 1;9(3):e01519-21. doi: 10.1128/Spectrum.01519-21 (PMC8635125; doi:10.1128/Spectrum.01519-21)
Supplement: SUPPLEMENTAL FILE 1 — Supplemental material. Download SPECTRUM01519-21_Supp_1_seq6.pdf, PDF file, 2.0 MB [file spectrum01519-21_supp_1_seq6.pdf]

Supplementary Information

Table S1. Susceptibility of antibiotic-resistant *Salmonella* Typhimurium strains to antibiotics.

(A) Reference strain and MDR clinical isolates

	Salmonella Typhimurium					
	ATCC 14028		NL08.10		0911R	
	MIC (mg/L)	R/I/S[a]	MIC (mg/L)	R/I/S[a]	MIC (mg/L)	R/I/S[a]
SW14	>64	-	>64	-	>64	-
Gentamicin	2	S	2	S	2	S
Tetracycline	8	I	>64	R	>64	R
Streptomycin	32	-	>64	-	>64	-
Chloramphenicol	16	I	32	R	32	R
Ampicillin	16	I	>64	R	>64	R

(B) Reference strain and ciprofloxacin-resistant clinical isolates

	Salmonella Typhimurium					
	ATCC 14028		SA10		SB10	
	MIC (mg/L)	R/I/S[a]	MIC (mg/L)	R/I/S[a]	MIC (mg/L)	R/I/S[a]
SW14	>64	-	>64	-	>64	-
Gentamicin	2	S	0.5	S	1	S
Ciprofloxacin	<0.125	S	4	R	4	R
Ofloxacin	<0.125	S	8	R	8	R

[a]The susceptibility of bacteria to antibiotics is divided into resistant (R), intermediate (I) and sensitive (S) categories according to CLSI criteria (1).

Table S2. Susceptibility of *Salmonella* Typhimurium to antibiotics in CAMH medium, LB medium and LPM medium.

	MIC (mg/L)		
	S. Typhimurium ATCC 14028		
	CAMH	LB	LPM
Vancomycin	2048	512	128
Ampicillin	4	4	1
Rifampicin	16	16	4

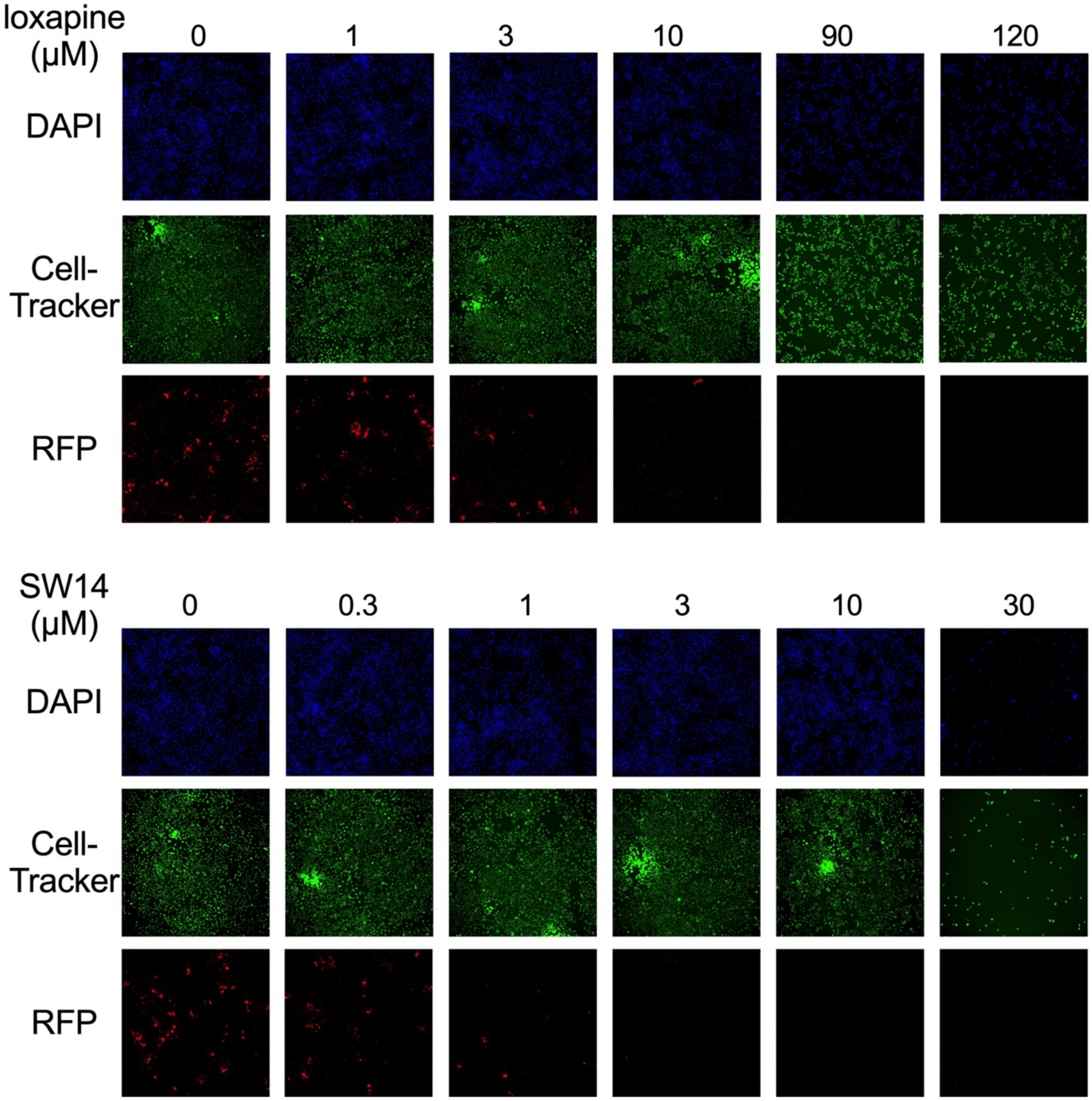

Figure S1. *Salmonella*-infected RAW264.7 cells were treated with loxapine (0 – 120 μM) or SW14 (0 – 30 μM) for 24 h followed by using DAPI and CellTracker to stain the nuclei and cytoplasm of infected cells, respectively. Bacteria were observed via detecting the fluorescence of RFP. The images were are representative of three independent experiments.

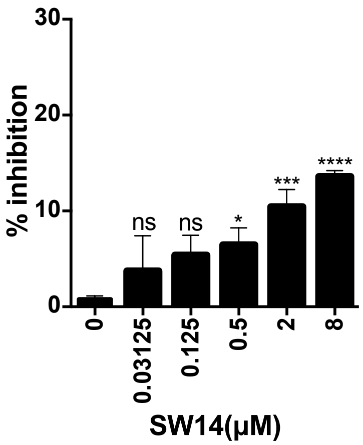

Figure S2. The accumulation of Hoechst 33342 in *S*. Typhimurium ATCC 14028 treated with escalated concentrations of SW14 for 1 h in the LPM medium was measured using a fluorescence microplate reader. The data are presented as the mean ± SD (n = 3 per group). ns: non-significant, $P > 0.05$; ***$P < 0.001$; ****$P < 0.0001$.

Hoechst 33342 accumulation assay

Overnight-grown *S.* Typhimurium cultured in LB medium was diluted 1:50 in LPM medium and incubated at 37°C for 3 h. The bacterial suspension was adjusted to an OD_{600} of 0.1 in fresh LPM medium supplemented with 2.5 μM Hoechst 33342 (Thermo Fisher Scientific) followed by treatment of mock (DMSO) or SW14 (0.03125 – 8 μM in DMSO) for 1 h. Bacterial cells which were treated with high temperature (90°C) for 10 min (heat-killed) rapidly accumulated Hoechst 33342 and served as positive control. Fluorescence was monitored on a SpectraMax M5 (Molecular devices) with a 355 nm excitation filter and 460 nm emission filter. The percent inhibition was calculated by the equation: $100 \times [(\text{SW14-treated} - \text{mock}) / (\text{Heat-killed} - \text{mock})]$.

References

1. CLSI. 2017. Performance Standards for Antimicrobial Susceptibility Testing — Eighteenth Informational Supplement: M100.
